# Healing of rotator cuff tendons using botulinum toxin A and immobilization in a rat model

**DOI:** 10.1186/s12891-016-0978-y

**Published:** 2016-03-15

**Authors:** Mohit N. Gilotra, Michael J. Shorofsky, Jason A. Stein, Anand M. Murthi

**Affiliations:** Department of Orthopaedics, University of Maryland School of Medicine, Baltimore, MD 21201 USA; Department of Orthopaedics and Sports Medicine, MedStar Union Memorial Hospital, Baltimore, MD 21218 USA

**Keywords:** Rotator cuff repair, Botulinum toxin A (Botox), Cast immobilization, Tendon healing, Rat model

## Abstract

**Background:**

We evaluated effects of botulinum toxin A (Botox) and cast immobilization on tendon healing in a rat model. Injection of Botox into rat supraspinatus was hypothesized to reduce muscle active force and improved healing.

**Methods:**

Eighty-four supraspinatus tendons were surgically transected and repaired in 42 Sprague-Dawley rats (transosseous technique). After repair, supraspinatus muscle was injected with saline or Botox (3 or 6 U/kg). Half the shoulders were cast-immobilized for the entire postoperative period; half were allowed free cage activity. Histology was examined at 2, 4, 8, and 12 weeks. A healing zone cross-sectional area was measured, and biomechanical testing of repair strength and tendon viscoelastic properties was conducted at 4 and 12 weeks.

**Results:**

Botox alone and cast immobilization alone exhibited increased ultimate load compared with controls (saline injection, no immobilization) at 4 weeks. No difference in ultimate load occurred between Botox-only and cast-only groups. At 12 weeks, the Botox (6 U/kg) plus cast immobilization group was significantly weakest (*p* < 0.05). A trend was shown toward decreased healing zone cross-sectional areas in casted groups.

**Conclusions:**

Supraspinatus Botox injection after rotator cuff repair might help protect the repair. However, cast immobilization plus Botox administration is harmful to rotator cuff healing in a rat tendon model.

## Background

Rotator cuff disorders account for 10 % of all shoulder pain in today’s aging population. Direct costs for shoulder treatment in the United States in the year 2000 reached US$7 billion, with no sign of this trend reversing [1]. Rates of re-rupture are as high as 90 %, depending on type of imaging, and some rotator cuff repairs do not heal [2]. When the rotator cuff does not heal after a repair, pain might be decreased initially but function and strength decline [2, 3].

Predictive factors that place a repair at risk include patient age, tear size, time from injury to repair, and postoperative activity level [4]. Various protocols of immobilization have been recommended to allow the rotator cuff to heal after repair without jeopardizing the long-term range of motion of the shoulder, but this tenet has been controversial [5]. Most surgeons have preferred initial immobilization, passive range of motion, and gradual progression to active assisted range of motion [6]. Because of patient compliance issues and biological challenges [7–10], optimizing the postoperative mechanical environment remains difficult.

Botulinum toxin A (Botox; Allergan, Inc., Irvine, CA) has been explored as a means of complete paralytic internal immobilization or “bioprotection” [11–13] in the setting of Achilles tendon and rotator cuff healing. Our purpose was to examine rotator cuff healing with intramuscular Botox injection in comparison with strict immobilization and with a non-immobilized saline control. We explored this question using two low doses of botulinum toxin A: 3 and 6 U/kg. The main objective was to learn whether a single Botox injection obviates the need for cast immobilization in a rodent rotator cuff injury model.

We hypothesized that botulinum toxin A injection into the rat supraspinatus muscle reduces the muscle’s active contractile force, allowing for improved healing at the supraspinatus footprint. Although no consensus has been reached regarding what “healing” truly is, we adapted the concept of a healing zone or the area of scar formation as healed to the greater tuberosity footprint.

## Methods

### Animal model

All animal procedures were approved by the Institutional Animal Care and Use Committee at the University of Maryland. Bilateral supraspinatus tendons of 42 male Sprague-Dawley skeletally immature rats (weight, 400–450 g) were surgically transected and repaired with a transosseous technique based on a well-established protocol [14–16]. Bilateral rotator cuff surgery has been standardized in multiple animal protocols [17, 18].

The animals were anesthetized with 5 % isoflurane induction and 2 % isoflurane maintenance delivered in an oxygen mixture via face masks. After clipping and sterile preparation were completed, a 1-cm incision was made over the posterior aspect of the shoulder. The posterolateral aspect of the deltoid was removed from the acromion. The supraspinatus was exposed with adduction of the extremity and forearm supination. A 5.0 Prolene suture (Ethicon, Somerville, NJ) was passed once through the supraspinatus for traction. The tendon was sharply transected with a No. 15 blade and firmly secured with completion of a locking stitch using a modified Mason-Allen technique. The anatomic footprint was burred at high speed, and a 0.5-mm drill hole was aimed in a cranial-caudal direction. The suture was passed through the drill hole and tied with the supraspinatus reapproximated to its anatomic footprint on the greater tuberosity. This rat injury model is analogous to an acute rotator cuff tear and not a chronic tear.

**Fig. 1:**
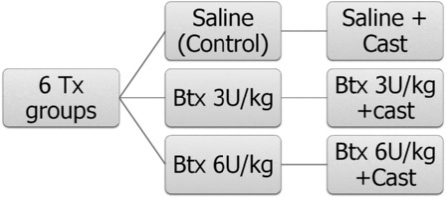
Chart clarifies treatment groups. Shoulders in three of six treatment (Tx) groups remained in casts during the entire postoperative period. Btx = Botox

Rat shoulders were randomly allocated by a random-number generator into six experimental groups (Fig. 1), ensuring one shoulder for a cast and one to be free from immobilization while housed in individual cages and allowed free cage activity. A power analysis (Stata 11; StataCorp LP, College Station, TX) that was conducted based on pilot work assessing ultimate load revealed that 60 shoulders were needed in total to provide 80 % power to detect a 30 % difference in ultimate load between groups with a *p* value of 0.05. One shoulder in each group was reserved for a strictly qualitative histological evaluation. Five shoulders in each group underwent biomechanic evaluation at 4 and 12 weeks, which was the main quantitative outcome of the study. The supraspinatus muscle was injected intraoperatively with either saline or botulinum toxin A after repair. The Botox dose administered was either 3 or 6 U/kg, based on previous small-animal bioprotection literature [11, 12]. Dilutions were standardized and volume was equal for all injections. The control group received saline injection into the supraspinatus and no cast immobilization. Half the shoulders remained in casts throughout the postoperative period. One shoulder of each animal was randomly allocated to an immobilized treatment group and the contralateral shoulder to a non-immobilized treatment group. All rats were thereby subjected to a cast and the same physiological stress. A unilateral shoulder was immobilized at 90° of forward flexion and 20° of abduction, which is a position of neutral weight bearing for the Sprague-Dawley rat. The paw and forearm were not included in the cast, and the animal was allowed to freely bear weight on both paws.

Analgesia (buprenorphine, 0.03 mg/kg, administered every 12 h) was provided by animal care staff during the immediate postoperative period. Animals were examined daily for signs of distress and cast discomfort. Rats underwent an obligatory cast change every other week. All cast changes were performed with the animals under anesthesia in a controlled fashion to prevent excessive passive manipulation of the shoulder.

Animals were euthanized at 2, 4, 8, and 12 weeks after rotator cuff repair. The entire supraspinatus muscle, tendon, and scar unit were harvested en bloc with the proximal humerus. Visual inspection revealed no gross, macroscopic repair failures. Histological analysis was conducted at all time points. Biomechanical testing was completed at the 4- and 12-week time points.

### Histological analysis

Specimens from each experimental group and all time points underwent histological analysis. Muscle tendon units were fixed in 4 % paraformaldehyde overnight and then underwent 2 days of gentle decalcification in 14 % ethylenediaminetetraacetic acid. The specimens were next embedded in paraffin, sectioned with a microtome into 3- to 4-μm sections, and dried. Samples were stained with toluidine blue for fibrocartilage detection and Masson trichrome blue for fibrous tissue detection. Hematoxylin and eosin testing was conducted to assess muscle cell morphology. Specimens were evaluated by a blinded pathologist specifically for fat formation in the muscle, collagen orientation in the repair, and the order of fibrocartilage proliferation.

### Biomechanical testing

Sixty shoulders from the 4- and 12-week groups were biomechanically tested for repair strength and tendon viscoelastic properties, as previously described [17–19]. The acromion and deltoid were initially removed, and subperiosteal dissection of the supraspinatus from its fossa was performed. The humerus and tendon were dissected free, and all excess soft-tissue constraints were removed from the humeral head. The humerus-tendon unit was stored in phosphate-buffered saline at 4 °C overnight for testing the next day. The insertion-site scar was measured with digital calipers, and an approximate area was determined by using an elliptical assumption: area = π × thickness × width. A three-dimensional scar volume was calculated based on the elliptical area times the scar length.

**Fig. 2:**
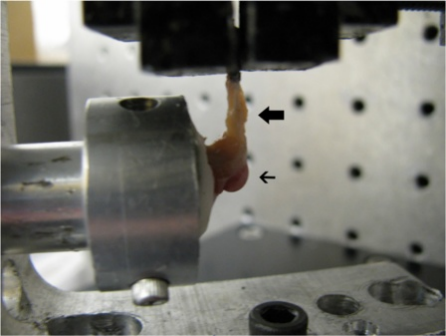
Harvested sample is shown. Supraspinatus tendon (*thick arrow*) is clamped at 90° to the humeral head (*thin arrow*) before load analysis

The distal portion of the humerus was potted in polymethylmethacrylate. The tendon was then fixed between two pieces of sand paper and was clamped at 90° to the supraspinatus tendon (Fig. 2). Biomechanical testing was conducted in a mini-materials testing machine (ElectroForce TestBench system; Bose Corporation, Eden Prairie, MN).

**Fig. 3:**
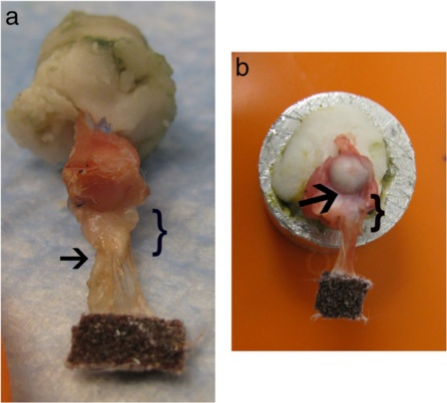
Areas of failure. **a** Failure at the tendon-scar junction. **b** Failure at the scar-bone interface. Bracket = scar, arrow = point of failure

The biomechanics loading protocol was based on a well-established model for testing rat rotator cuff repair [12, 16]. Specimens were initially preloaded to 0.2 N, and then 5 cycles of preconditioning were applied to a strain of 5 %. The strain measurement was based on the distance from the clamp to the tendon insertion. The conditioning rate remained a constant 0.1 % per second for examination of viscoelastic properties. The specimen was then stress-relaxed at 5 % strain for 5 min. Tendons were loaded to failure at a constant rate (0.1 % per second). Stress was calculated as tensile force applied ÷ initial calculated cross-sectional area. Stiffness was same force ÷ linear displacement, whereas the tendon-scar modulus was extrapolated from the linear portion of the stress-strain curve. Ultimate load and stress were determined based on the point of failure. Anatomic location of failure was recorded as one of three categories: tendon-scar interface, scar-bone interface, or indeterminate (Fig. 3).

### Statistical analysis

Biomechanical results were subjected to a two-factor analysis of variance and a Tukey post hoc test to allow for multiple-group analysis with all groups compared with only the non-treated (no Botox or cast) group. Statistical significance was set at *p* < 0.05. Anatomic site of failure was strictly observational, and statistical analysis was not conducted on histological results because they were qualitative.

## Results

Before euthanization, all animals survived the treatment in good health. No adverse events occurred.

### Histology

Muscle atrophy was observed on gross examination; fatty infiltration was noted on a cellular level. The two groups subjected to both Botox injection and immobilization exhibited wasting in the supraspinatus fossa. The findings were corroborated with histological findings of extensive fat infiltration. Cast immobilization alone at 12 weeks echoed similar results: atrophy shown on gross examination and fatty infiltration on histological examination. At 2 and 4 weeks, Botox-only groups had no gross supraspinatus atrophy but some fatty infiltration was revealed by histological examination.

**Fig. 4:**
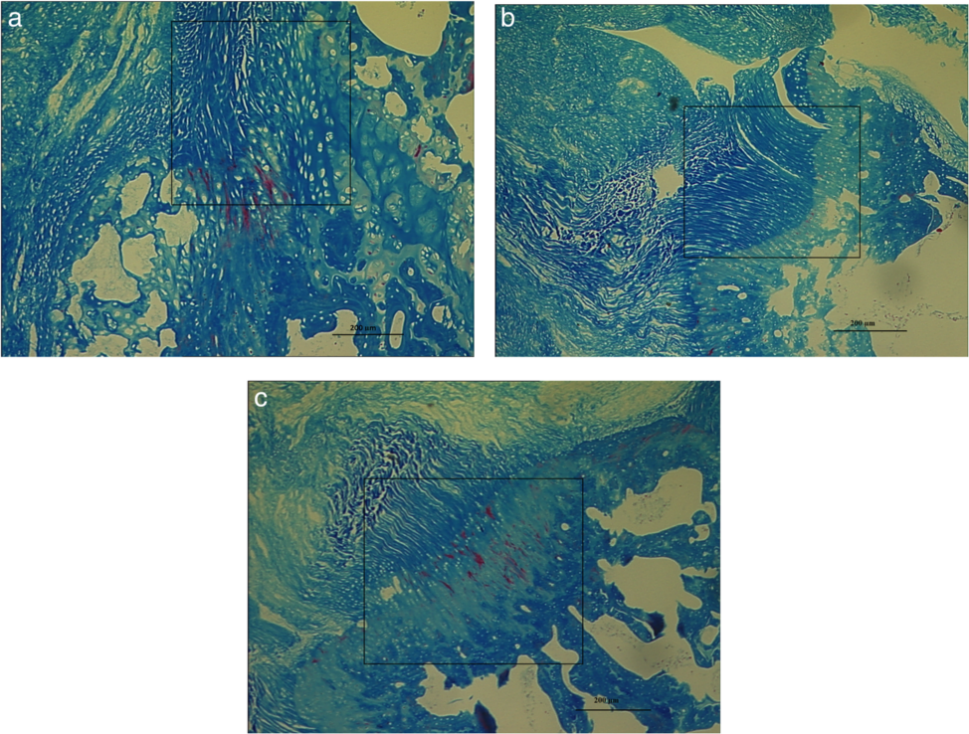
Comparison of photomicrographs. **a** Non-immobilized saline control. **b** Botox 6 U/kg alone. **c** Cast alone. Masson’s trichrome blue stain (original magnification, 10×) of scar at 4 weeks shows a disorganized scar for the non-immobilized control and a more organized scar for the Botox-only and cast-only specimens. Rectangles indicate regions of interest

At 8 and 12 weeks, however, the Botox-only groups had no fatty findings. At 2 weeks, all specimens demonstrated a disorganized fibrocartilage scar at the tendon-bone interface. At 8 and 12 weeks, no difference was observed in scar organization among all groups. At 4 weeks, shoulders subjected to Botox only, cast immobilization only, or Botox plus cast immobilization showed a more linear formation of collagen compared with the saline control group (Fig. 4).

### Biomechanics

**Fig. 5:**
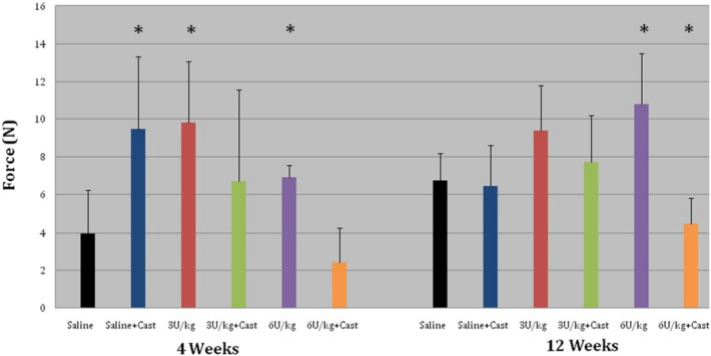
Bar chart depicts force. Ultimate load at 4 and 12 weeks. At 4 weeks, Botox-only and cast-only groups were significantly stronger than the control group. At 12 weeks, Botox 6 U/kg was the strongest and 6 U/kg with cast immobilization was the weakest. Asterisk = significant values (*p* < 0.05) after Tukey post hoc test. Error bars correspond to standard deviations

The ultimate load of the repaired supraspinatus tendon in the 4-week samples showed that the tendons receiving either dose of Botox alone and those receiving cast immobilization alone were significantly stronger than those in the control group (*p* < 0.05). At 12 weeks, increased load to failure was shown for the Botox-only (6 U/kg dose) group compared with the cast-only group and the saline-only control group (Fig. 5). Botox (6 U/kg) in combination with cast immobilization was significantly the weakest group at both time points (*p* < 0.05).

No statistical difference was observed in the healing zone cross-sectional scar area or scar volume across groups. The cast-only group at 12 weeks tended to have decreased scar volume compared with the control group.

**Fig. 6:**
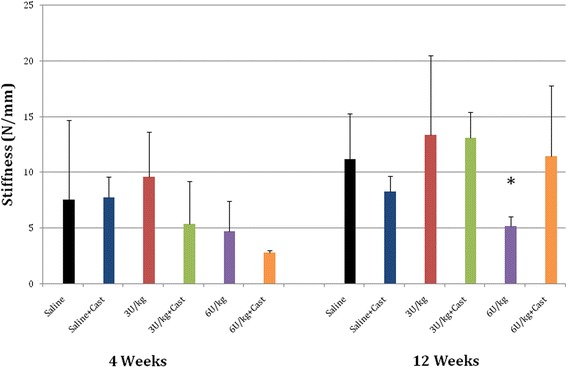
Bar chart depicts stiffness. Shoulders that received 6 U/kg Botox were least stiff at 12 weeks compared with control group shoulders. Asterisk = significant values (*p* < 0.05) after Tukey post hoc test. Error bars correspond to standard deviations

No difference in stiffness was observed at 4 weeks (Fig. 6). Shoulders that received 6 U/kg Botox were the least stiff compared with the 12-week groups and were significantly different from control group shoulders at the 12-week time point (*p* < 0.05). At 4 weeks, 21 (88 %) of 24 repairs in the cast-only and Botox-only groups combined failed at the scar-bone interface. All the saline control group repairs, however, failed at the tendon-scar junction. At 12 weeks, gross inspection showed that all specimens failed at the scar-bone interface.

## Discussion

We assessed whether intraoperative Botox injection optimizes the early postoperative mechanical environment in a rat rotator cuff healing model. We paralyzed the supraspinatus with botulinum toxin A after rotator cuff injury and repair. We used a lower dose of Botox than that used in previous studies [11, 12] to allow some load at the repair site. Half the shoulders were additionally treated with immobilization, and the other half were allowed free cage activity. Cast immobilization in combination with 6 U/kg Botox paralysis impaired the repair quality. Botox treatment combined with free cage activity provided repair strength similar to that provided by immobilization. Some force at the repair site is necessary for tendon healing. Early active motion has been shown to be beneficial in preserving hand motion for flexor tendon repairs without hampering the healing process [20]. These positive effects of early motion result in a decrease in adhesion formation but might not improve the quality of the healing tissue [21]. Early motion also has importance in enthesis healing. Increasing static tension has proved to be beneficial at the knee in medial collateral ligament healing [22].

Immediate passive motion in rotator cuff models has been shown to have a detrimental effect. Peltz et al. [23] examined immediate passive motion versus strict immobilization after rat rotator cuff repair. Although no difference was observed in rotator cuff mechanical properties, the immediate motion group showed decreased range of motion, likely from increased subacromial scar formation. Shoulders that were immobilized exhibited mechanical properties superior to those of exercised shoulders [24].

Tendon healing benefits from temporary immobilization and responds to early motion. Bedi et al. [25] showed that delayed loading in anterior cruciate ligament reconstruction in a Sprague-Dawley rat model was the optimum mechanical environment, better than both prolonged immobilization and immediate loading. Our results echo this “happy medium” theory. In our study, at 12 weeks, a single intraoperative injection of Botox provided the best mechanical environment. Botulinum toxin A provides early internal immobilization through reversible chemical paralysis. It is a natural poison that inhibits presynaptic acetylcholine release. Most of the effect of botulinum toxin A decreases after the first few weeks [26]. The repair site is then subjected to some passive motion during the early postoperative period and then active and passive motion while the rotator cuff tendons remodel.

Botulinum toxin impacts the local mechanical environment and might also affect local biology factors. Recent literature has focused on the biology as the cause of tenopathy and tendon re-tear rather than the stiffness of the repair construct or other mechanical theories [27, 28]. Botox has been used clinically for chronic tendinopathies, including lateral epicondylitis [29, 30]. Multiple mechanisms have been proposed, including reversible paralysis of the common extensor to allow microtrauma to heal versus modulation of local pain receptors. The proposed mechanism of Botox-assisted rotator cuff healing is unknown. Şahin et al. [31] found improvements in gait analysis after intramuscular Botox injection after cuff repair. It is unknown, however, whether this is a function of anti-nociceptive effects of Botox or improved cuff healing.

Bioprotection with Botox has been studied in various tendon-to-bone healing models. Ma et al. [11] explored the use of Botox to decrease the initial active force of the rat gastrocnemius muscle after an Achilles tendon repair. Twitch and tetanus contractions decreased 25 to 50 % during the initial month but returned to baseline measurements after 6 months. The spontaneous rupture rate of repaired tendons was significantly lower at 1 and 3 weeks after repair (*p* = 0.007). Our acute model did not evaluate the early failure rate of the rotator cuff repair but rather the mechanical properties of the healing scar. This is a limitation of the acute rat rotator cuff model as opposed to the Achilles repair model. No re-tears occur in the rat rotator cuff because it heals spontaneously and quickly.

In 2009, botulinum toxin A-assisted bioprotection for rotator cuff repair was assessed in a rat rotator cuff injury model [12]. Galatz et al. [12] injected 9 U/kg Botox into the supraspinatus muscle of rats after rotator cuff repair. This group was compared with a cast immobilization control group and a group treated with both Botox and immobilization. The combination of Botox and casting proved to be detrimental to rotator cuff repair, especially at 8 weeks, whereas immobilization alone was superior at both 4 and 8 weeks.

We observed similar discouraging findings in a study of chronic rabbit rotator cuff tears treated with intramuscular Botox [32]. The atrophic supraspinatus muscle was unable to overcome the denervating insult of Botox, leading to an inferior repair. In the present study, we extended one time point to 12 weeks and added lower dosage groups of 6 U/kg and 3 U/kg to better judge recovery after botulinum toxin paralysis. We found similar results when combining paralysis with immobilization. Even with strict immobilization, some eccentric contraction of the rotator cuff occurs, allowing for some beneficial force at the repair site. The addition of Botox paralysis decreases this force, leading to decreased repair quality.

Botulinum toxin A alone, however, showed some improved rotator cuff biomechanical properties in our study. We used low Botox doses of 3 and 6 U/kg to reduce the supraspinatus muscle active force. In addition, we extended our final end point to 12 weeks, allowing for the rotator cuff repair to remodel further after the Botox effect continued to wean. The maximal effect of the injection, however, occurs approximately 1 week after injection. In one study, the intramuscular injection was administered 1 week before the rat rotator cuff injury and repair. Histological organization of the scar was improved, especially tears subjected to increased load [33].

In our study, histological examination of the repair site showed no difference at the cellular response level. The only appreciable histological difference was the scar orientation at 4 weeks. Groups treated with some kind of immobilization (cast or Botox) exhibited a more linear scar response. The scar orientation at 4 weeks matched the location of load to failure results. At 4 weeks, failure in the saline control group occurred at a lower load and at the tendon-scar junction. Failure in the cast-only and Botox-only groups occurred at higher loads at the scar-bone site. Biomechanical failure at the scar-bone interface might be a sign of early remodeling when compared with failure at the tendon-scar interface. At 12 weeks, a majority of the constructs showed a linear scar response and failure at the scar-bone site.

This study used a well-established rat rotator cuff injury model that included both cast immobilization groups and a saline-only control group. The biomechanical and histological protocols have been well described [34], rendering our results comparable with those of other rodent models. We studied multiple treatment groups and varied the dose of botulinum toxin A. In addition, we recorded the site of failure, which has not been previously described in the literature. Our study provides important information in the setting of an acute rotator cuff tear model.

A limitation of this study was the acute repair model in a quadruped. Rotator cuff repairs with healing difficulties typically are chronic in nature. It is possible that optimizing the mechanical environment with Botox might not overcome the poor biological condition of chronic rotator cuff degeneration. In addition, rotator cuff muscles in the setting of a chronic tear exhibit baseline fatty degeneration and atrophy [35, 36]. The additive paralysis effect of Botox in this setting is unknown and might cause a more deleterious fatty response, as previously shown [32]. The exact paralytic effect of Botox on a small rat supraspinatus muscle is unknown without specific functional muscle testing. Therefore, we are unable to quantify the paralyzed “load” exacted on the repair site.

The literature on the topic of botulinum toxin-assisted rotator cuff repair is confusing. Multiple factors need to be considered, including timing of injection, acute versus chronic repair, combining chemical paralysis with immobilization, and the species of the animal model [12, 31–33]. Botox might have its best effect when injected before an acute repair with minimal immobilization. Testing in an animal model that exhibits a clinically relevant incidence of re-tear would help to resolve a current controversy in the literature.

## Conclusions

Botulinum toxin A injection into the supraspinatus might obviate the need for postoperative immobilization. Chemical paralysis in addition to cast immobilization reduces load at the repair site and is harmful to rotator cuff repair. Clinical implications include potentially decreased postoperative immobilization time, better long-term functional outcomes, improved compliance, and reduced stiffness with increased protection of tendon repair sites.
